# Protocol for a randomized, placebo-controlled, double-blind clinical trial investigating sacral neuromodulation for neurogenic lower urinary tract dysfunction

**DOI:** 10.1186/1471-2490-14-65

**Published:** 2014-08-13

**Authors:** Stephanie C Knüpfer, Martina D Liechti, Livio Mordasini, Dominik Abt, Daniel S Engeler, Jens Wöllner, Jürgen Pannek, Bernhard Kiss, Fiona C Burkhard, Marc P Schneider, Elena Miramontes, Alfons G Kessels, Lucas M Bachmann, Thomas M Kessler

**Affiliations:** 1 Neuro-Urology, Spinal Cord Injury Center & Research, University of Zürich, Balgrist University Hospital, Forchstrasse 340, 8008 Zürich, Switzerland; 2 Department of Urology, Cantonal Hospital St. Gallen, St. Gallen, Switzerland; 3 Neuro-Urology, Swiss Paraplegic Center, Nottwil, Switzerland; 4 Department of Urology, University of Bern, Bern, Switzerland; 5 Department of Clinical Epidemiology and Medical Technology Assessment, Maastricht University Medical Center, Maastricht, The Netherlands; 6 Medignition Inc., Research Consultants, Zug, Switzerland

**Keywords:** Urinary bladder, Neurogenic lower urinary tract dysfunction, Sacral neuromodulation, Randomized, Placebo-controlled, Double-blind trial

## Abstract

**Background:**

Sacral neuromodulation has become a well-established and widely accepted treatment for refractory non-neurogenic lower urinary tract dysfunction, but its value in patients with a neurological cause is unclear. Although there is evidence indicating that sacral neuromodulation may be effective and safe for treating neurogenic lower urinary tract dysfunction, the number of investigated patients is low and there is a lack of randomized controlled trials.

**Methods and design:**

This study is a prospective, randomized, placebo-controlled, double-blind multicenter trial including 4 sacral neuromodulation referral centers in Switzerland. Patients with refractory neurogenic lower urinary tract dysfunction are enrolled. After minimally invasive bilateral tined lead placement into the sacral foramina S3 and/or S4, patients undergo prolonged sacral neuromodulation testing for 3–6 weeks. In case of successful (defined as improvement of at least 50% in key bladder diary variables (i.e. number of voids and/or number of leakages, post void residual) compared to baseline values) prolonged sacral neuromodulation testing, the neuromodulator is implanted in the upper buttock. After a 2 months post-implantation phase when the neuromodulator is turned ON to optimize the effectiveness of neuromodulation using sub-sensory threshold stimulation, the patients are randomized in a 1:1 allocation in sacral neuromodulation ON or OFF. At the end of the 2 months double-blind sacral neuromodulation phase, the patients have a neuro-urological re-evaluation, unblinding takes place, and the neuromodulator is turned ON in all patients. The primary outcome measure is success of sacral neuromodulation, secondary outcome measures are adverse events, urodynamic parameters, questionnaires, and costs of sacral neuromodulation.

**Discussion:**

It is of utmost importance to know whether the minimally invasive and completely reversible sacral neuromodulation would be a valuable treatment option for patients with refractory neurogenic lower urinary tract dysfunction. If this type of treatment is effective in the neurological population, it would revolutionize the management of neurogenic lower urinary tract dysfunction.

**Trial registration:**

*Trial registration number:*http://www.clinicaltrials.gov; Identifier: NCT02165774.

## Background

Neurogenic lower urinary tract dysfunction (LUTD) is highly prevalent and affects the lives of millions of people worldwide. It has a major impact on quality of life and, besides the debilitating manifestations for patients, it also imposes a substantial economic burden for every healthcare system. Neurogenic LUTD is a challenge because all available treatment modalities (i.e. conservative, minimally invasive and surgical therapies) may either fail or be invasive causing considerable complications and/or side effects.

Sacral neuromodulation (SNM) [1] has become a well-established and widely accepted treatment for patients with refractory LUTD such as non-obstructive chronic urinary retention, urgency frequency syndrome, and urgency incontinence [2-6] and it has been incorporated into the guidelines of the European Association of Urology (EAU) ( http://www.uroweb.org), the International Consultation on Incontinence (ICI) [7], and the National Institute for Health and Clinical Excellence (NICE) ( http://www.nice.org.uk). Originally, SNM was not considered an option for neurogenic LUTD but some studies suggested that it is also effective in neurological patients [3,8]. Considering that SNM is minimally invasive and completely reversible, it is of great interest whether this is a valuable treatment option for patients with neurogenic LUTD. In a recent systematic review and meta-analysis [9], we found that there is evidence indicating that SNM may be effective and safe for the treatment of this group of patients. However, the number of investigated patients is low with high between-study heterogeneity and there is a lack of randomized controlled trials [9].

We therefore designed a prospective, randomized, placebo-controlled, double-blind multicenter clinical trial to assess the efficacy and safety of SNM for treating patients with neurogenic LUTD. The study hypothesis is that in patients with refractory neurogenic LUTD, SNM leads to an at least 35% increase in success rate as compared to placebo (i.e. sham) stimulation within 2 months, i.e. SNM is considerably more effective than placebo (i.e. sham) stimulation.

## Methods and design

### Study design

This study is a prospective, randomized, placebo-controlled, double-blind multicenter trial including 4 SNM referral centers in Switzerland: Neuro-Urology, Spinal Cord Injury Center & Research, University of Zürich, Balgrist University Hospital, Zürich; Department of Urology, Cantonal Hospital St. Gallen, St. Gallen; Neuro-Urology, Swiss Paraplegic Center Nottwil, Nottwil; Department of Urology, University of Bern, Bern.

**Figure 1:**
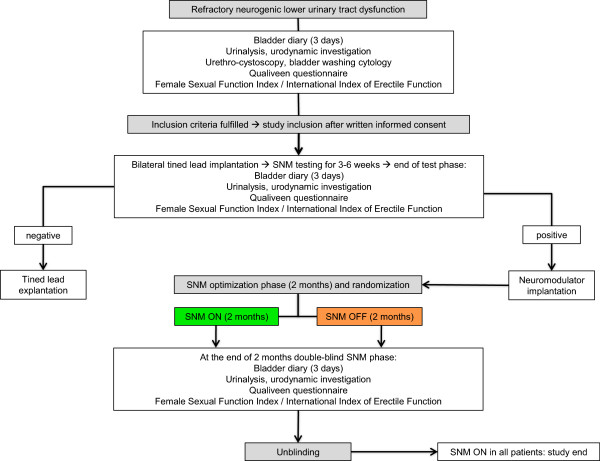
**Flowchart of the sacral neuromodulation (SNM) trial.** SNM: sacral neuromodulation.

In case of successful prolonged SNM, the neuromodulator is implanted and patients are randomized using a computer program considering 3 strata according to the neuro-urological diagnosis, i.e. a) urgency frequency syndrome and/or urgency incontinence, b) chronic urinary retention, and c) combination of urgency frequency syndrome and/or urgency incontinence and chronic urinary retention. After a 2 months SNM optimization phase following neuromodulator implantation, the neuromodulator is turned ON or OFF in a 1:1 allocation by an investigator not involved in the assessment of the clinical outcome.The Figure 1 gives an overview of the procedures that the patients will undergo during the course of the study.

### Study population and recruitment

According to the inclusion and exclusion criteria (Table 1), we will investigate patients with refractory neurogenic LUTD. The following variables will be considered: gender, age, neurological disease, duration of neurological disease, previous treatment, bladder diary variables, urinalysis, urethro-cystoscopy, bladder washing cytology, urodynamic investigations, and questionnaires, i.e. Female Sexual Function Index (FSFI) [10]/International Index of Erectile Function (IIEF) [11] and Qualiveen [12].

**Table 1:** Inclusion and exclusion criteria for patients with refractory neurogenic lower urinary tract dysfunction (LUTD)

**Inclusion criteria**	**Exclusion criteria**
▪ Age >18 years	▪ Age <18 years
▪ Refractory neurogenic LUTD	▪ Non-neurogenic LUTD
▪ Urgency frequency syndrome and/or urgency incontinence refractory to antimuscarinics (pharmacotherapy for at least 4 weeks with at least 2 antimuscarinics)	▪ Botulinum toxin injections into the detrusor and/or urethral sphincter in the last 6 months
▪ Chronic urinary retention refractory to alpha-blocker (pharmacotherapy with an alpha-blocker for at least 4 weeks)	▪ Pregnancy or breast feeding
▪ Combination of urgency frequency syndrome and/or urgency incontinence refractory to antimuscarinics (pharmacotherapy for at least 4 weeks with at least 2 antimuscarinics) and chronic urinary retention refractory to alpha-blocker (pharmacotherapy with an alpha-blocker for at least 4 weeks)	▪ Individuals especially in need of protection (according to Research with Human Subjects published by the Swiss Academy of Medical Sciences ( http://www.samw.ch/en/News/News.html))
▪ Written informed consent	▪ No written informed consent

### Determination of sample size

We are planning a study of independent cases and controls with 1 control per case. Prior data indicate that the (spontaneous) success rate among controls is 0.15. If the true success rate for experimental subjects (SNM ON) is at least 0.5, we will need to study 27 experimental subjects (SNM ON) and 27 control subjects (SNM OFF) to be able to reject the null hypothesis that the failure rates for experimental (SNM ON) and control subjects (SNM OFF) are equal with probability (power) 0.8. The type I error probability associated with this test of this null hypothesis is 0.05. Taking into account potential drop-outs, we will include 30 patients per group.

### Study location and partners

▪ Neuro-Urology, Spinal Cord Injury Center & Research, University of Zürich, Balgrist University Hospital, Zürich, Switzerland

▪ Department of Urology, Cantonal Hospital St. Gallen, St. Gallen, Switzerland

▪ Neuro-Urology, Swiss Paraplegic Center, Nottwil, Switzerland

▪ Department of Urology, University of Bern, Switzerland

▪ Department of Clinical Epidemiology and Medical Technology Assessment, Maastricht University Medical Center, Maastricht, The Netherlands

▪ Medignition Inc., Research Consultants, Zug, Switzerland

### Investigations

In case the patients with refractory neurogenic LUTD fulfill the study inclusion criteria following neuro-urological evaluation (bladder diary for at least 3 days, urinalysis, urodynamic investigation, urethro-cystoscopy and bladder washing cystology, Qualiveen questionnaire [12], FSFI [10]/IIEF [11], they are included after providing written informed consent. The Figure 1 gives an overview of the procedures that the patients will undergo during the course of the study. After minimally invasive bilateral tined lead placement into the sacral foramina S3 and/or S4 (stage one), patients undergo prolonged SNM testing for 3–6 weeks completing a bladder diary to assess the response to treatment. In accordance with the literature [5,6], an improvement of at least 50% in the key bladder diary variables (i.e. number of voids and/or number of leakages, post void residual) compared to the baseline values is considered a positive test and an indication for neuromodulator implantation. At the end of the test phase, the patients have a neuro-urological re-evaluation (bladder diary for at least 3 days, urinalysis, urodynamic investigation, Qualiveen questionnaire [12], FSFI [10]/IIEF [11]). In case of negative prolonged SNM testing, the tined leads are explanted. In case of successful prolonged SNM testing, the neuromodulator is generally implanted in the upper buttock (rarely in the anterior abdominal wall) (stage two). After neuromodulator implantation, each patient has a 2 months phase when the neuromodulator is turned ON using sub-sensory threshold stimulation (SNM optimization phase) to optimize the effectiveness of neuromodulation by determining the most effective stimulation parameters (choice of stimulation electrodes, intensity of stimulation) for each patient. At the end of the SNM optimization phase, patients are randomized in a double-blind parallel design to SNM ON or OFF. During an outpatient visit, neuromodulation parameters and bladder diary parameters are checked and the neuromodulator is turned ON or OFF by an investigator not involved in assessment of the clinical outcome. Considering that sub-sensory stimulation is used for SNM, the patients do not feel if the stimulation is ON or OFF. At the end of the 2 months double-blind SNM phase, the patients have a neuro-urological re-evaluation (bladder diary for at least 3 days, urinalysis, urodynamic investigation, Qualiveen questionnaire [12], FSFI [10]/IIEF [11]). During this visit, unblinding takes place and the neuromodulator is turned ON in all patients.

### Safety

The investigators will inform the patients, the study monitoring board, and the ethics committee if it becomes evident that the disadvantages of participation may be significantly greater than was foreseen in the research proposal. The study will be suspended pending further review by the study monitoring board, except insofar as suspension would jeopardize the patients’ health. The investigators will take care that all patients are kept informed.

Adverse events will be assessed and categorized according to the National Cancer Institute Common Terminology Criteria for Adverse Events (CTCAE) version 4 in grade 1 to 5 ( http://ctep.cancer.gov/protocolDevelopment/electronic_applications/ctc.htm). All adverse events will be followed until they have abated, or until a stable situation has been reached. Depending on the event, follow-up may require additional tests or medical procedures as indicated, and/or referral to the general physician or a medical specialist.

In the case of withdrawal of consent to participate in the study, all possible efforts will be made to convince the patient to continue to have safety follow-up evaluations.

In the event one of the following situations arises among treated patients during the conduct of the study, the study will be temporarily suspended and a comprehensive safety review conducted evaluating if the study has to be terminated prematurely:

▪ Any death secondary to rapid unexpected progression of an underlying medical condition.

▪ Severe clinical or neurological deterioration in more than one subject.

▪ Any other serious adverse event determined by the study monitoring board to be a reason to suspend the study.

### Study outcome measures

Primary: Success of SNM: Defined in accordance with the literature [5,6] as improvement of at least 50% in the key bladder diary variables (i.e. number of voids and/or number of leakages, post void residual) compared to the baseline values (i.e. patients with urgency frequency syndrome and/or urgency incontinence: at least 50% decrease in number of voids and/or number of leakages; patients with chronic urinary retention: at least 50% decrease in post void residual; patients with a combination of urgency frequency syndrome and/or urgency incontinence and chronic urinary retention: at least 50% decrease in number of voids and/or number of leakages, and/or at least 50% decrease in post void residual).

Secondary: A) Adverse events: Categorization according to the National Cancer Institute Common Terminology Criteria for Adverse Events (CTCAE) version 4 in grade 1 to 5 ( http://ctep.cancer.gov/protocolDevelopment/electronic_applications/ctc.htm).

B) Urodynamic parameters: cystometric capacity (mL), compliance (mL/cmH_2_O), detrusor overactivity (if yes: bladder volume (mL) at detrusor overactivity, maximum detrusor pressure amplitude (cmH_2_O), detrusor leak point pressure (cmH_2_O)), maximum detrusor pressure (cmH_2_O), detrusor pressure at maximum flow rate (cmH_2_O), maximum flow rate (mL/s), voided volume (mL), post void residual (mL), pelvic floor electromyographic activity (normal/detrusor sphincter dyssynergia).

C) Questionnaires, i.e. Qualiveen and FSFI/IIEF.

D) Costs of SNM.

### Data analysis

#### Statistics

Interval scaled variates will be summarized with means and standard deviations (SD) or medians and interquartile ranges where appropriate. Dichotomous variates will be described as ratios and percentages.

#### Univariate analysis

T-tests will be used to compare means between groups and chi-squared tests to compare dichotomous variables.

#### Multivariate analysis

To adjust for unequal distribution of parameters at baseline, multivariate regression models, linear models in case of an interval scaled outcome and logistic regression in case of a dichotomous outcome will be performed.

### Ethics and dissemination

This trial will be performed in accordance with the World Medical Association Declaration of Helsinki [13], the guidelines for Good Clinical Practice [14] and the guidelines of the Swiss Academy of Medical Sciences [15]. Handling of all personal data will strictly comply with the federal law of data protection in Switzerland [16]. The trial has been registred at clinicaltrials.gov (www.clinicaltrials.gov/ct2/show/NCT02165774).

## Discussion

First-line treatment for neurogenic LUTD includes antimuscarinics and some form of catheterization if necessary, preferably intermittent self-catheterization [17]. However, the treatment effect is often unsatisfactory, so that other options have to be considered, including onabotulinumtoxinA injections into the detrusor [18] or more invasive procedures such as bladder augmentation or urinary diversions. Thus, it is of utmost importance to know whether the minimally invasive and completely reversible SNM, a well established and widely accepted therapy for refractory non-neurogenic LUTD, would be a valuable treatment option for patients with refractory neurogenic LUTD. In addition, SNM may enable voiding without intermittent catheterization, the standard technique for chronic neurogenic urinary retention today. As a significant number of patients cannot perform this technique due to the underlying neurological disorder, SNM may not only prevent major surgery but also life-long treatment with indwelling catheters, which are related to significant long-term complications.

Assessing efficacy and safety of SNM for neurogenic LUTD, it is essential to be aware of the fact that these patients usually have undergone multiple failed previous treatments. In the case that SNM is also effective in the neurological population, this would have major implications for daily practice and would completely revolutionize the management of neurogenic LUTD.

This trial is multidisciplinary and will significantly influence all involved disciplines, i.e. neuro-urology, urology, and neurology. Especially in neurology, this project will increase the awareness of LUTD in neurological disorders and the related effective treatment options including SNM.

### Ethics approval

This study has been approved by the local ethics committees (Kantonale Ethikkommission Zürich KEK-ZH-Nr. 2011–0048, St. Gallen KEK-SG-Nr. 12/069, Luzern KEK-LU-Nr. 12047, Bern KEK-BE-Nr. 094/12).

## Abbreviations

CTCAE: Common Terminology Criteria for Adverse Events; EAU: European Association of Urology; FSFI: Female Sexual Function Index; ICI: International Consultation on Incontinence; IIEF: International Index of Erectile Function; LUTD: Lower urinary tract dysfunction; NICE: National Institute for Health and Clinical Excellence; SNM: Sacral neuromodulation.

## Competing interests

The authors declare that they have no competing interests.

## Authors’ contributors

TMK, DSE, AGK, and LMB created the study design. SCK and TMK drafted the manuscript. MDL, LM, DA, DSE, JW, JP, BK, FCB, MPS, EM, AGK, and LMB critically reviewed the manuscript. TMK obtained the funding of this study. All the authors read and approved the final manuscript.

## Pre-publication history

The pre-publication history for this paper can be accessed here:

http://www.biomedcentral.com/1471-2490/14/65/prepub
